# Modelling of Radiological Health Risks from Gold Mine Tailings in Wonderfonteinspruit Catchment Area, South Africa

**DOI:** 10.3390/ijerph13060570

**Published:** 2016-06-07

**Authors:** Manny Mathuthu, Caspah Kamunda, Morgan Madhuku

**Affiliations:** 1 Center for Applied Radiation Science and Technology, North West University (Mafikeng), P.Bag X2046, Mmabatho 2735, South Africa; Manny.Mathuthu@nwu.ac.za; 2 iThemba LABS, National Research Foundation, Private Bag X11, WITS 2050, South Africa; madhuku@tlabs.ac.za

**Keywords:** RESRAD-OFFSITE code, radionuclides, mine tailings, radiation dose, cancer morbidity risk, activity concentration

## Abstract

Mining is one of the major causes of elevation of naturally-occurring radionuclide material (NORM) concentrations on the Earth’s surface. The aim of this study was to evaluate the human risk associated with exposure to NORMs in soils from mine tailings around a gold mine. A broad-energy germanium detector was used to measure activity concentrations of these NORMs in 66 soil samples (56 from five mine tailings and 10 from the control area). The RESidual RADioactivity (RESRAD) OFFSITE modeling program (version 3.1) was then used to estimate the radiation doses and the cancer morbidity risk of uranium-238 (^238^U), thorium-232 (^232^Th), and potassium-40 (^40^K) for a hypothetical resident scenario. According to RESRAD prediction, the maximum total effective dose equivalent (TEDE) during 100 years was found to be 0.0315 mSv/year at year 30, while the maximum total excess cancer morbidity risk for all the pathways was 3.04 × 10^−5^ at year 15. The US Environmental Protection Agency considers acceptable for regulatory purposes a cancer risk in the range of 10^−6^ to 10^−4^. Therefore, results obtained from RESRAD OFFSITE code has shown that the health risk from gold mine tailings is within acceptable levels according to international standards.

## 1. Introduction

Naturally-occurring radioactive materials (NORMs) are a common occurrence in our environment since the formation of the Earth. These could be of cosmic, terrestrial, or internal origin [1]. They are generally available in the environment at levels that are not potentially harmful to human health. NORMs account for up to 85% of the annual dose exposure received by the world population [2]. In certain cases, anthropogenic activities, such as mining, have produced wastes that contain radiation above background levels in the environment, a situation that has been of major concern for radiation protection [3]. Mining can contaminate soils over a large area through radiation exposure and other environmental contaminants [4]. This eventually affects humans through different radiation exposure pathways, either external or internal (*i.e.*, ingestion and inhalation pathways).

NORMs such as ^238^U, ^232^Th, and ^40^K provide significant sources of human exposure to ionizing radiation [5]. One of the greatest legacies of South Africa’s extensive mineral deposits has been its vast infrastructure and wealth. However, this has left another, more troubling, legacy of environmental contamination from the many years of mining. The epicentre of the problem is in the southwest of Johannesburg where gold mining has been carried out for more than a century in an area called the Wonderfonteinspruit Catchment Area (WCA) [6]. Gold and uranium mining in the WCA has taken place from more than 120 mines, with extraction of 43,500 tonnes of gold in one century and 73,000 tonnes of uranium between 1953 and 1995 [6]. This uranium is the principal contaminant of concern within the gold mining areas of the WCA. It is reported that gold-bearing ores may contain almost ten times the amount of uranium than gold [7]. It is believed that the uranium content in many gold mine tailings exceeds the exclusion limit for regulation by the National Nuclear Regulator [8]. With mine tailings littered everywhere within the WCA, this produces large quantities of uranium in the area, inducing a radiological burden to man and possible damage to agricultural land and water resources.

Although a number of radiological studies have been carried out in the WCA, very little concrete data is available for the gold mining area in question. The extent of the possible risk of these radionuclides to the health of the population in the study area has also not been fully documented. As a result, radiological measurements were conducted on gold mine tailings in order to estimate the human risk caused by these radionuclides for a hypothetical resident scenario with the help of the RESidual RADioactivity (RESRAD) computer program (Argonne, Lemont, IL, USA). Exposure scenarios are patterns of human activity that can affect the release of radioactivity from the contaminated zone and the amount of exposure received at the exposure location. The hypothetical resident scenario includes all environmental pathways for on-site or near-site exposure that results in the highest predicted lifetime dose [9].

## 2. Materials and Methods

### 2.1. The Study Area

The study area is located in the West Wits line (Far West Rand) Goldfield of the lower central part of Wonderfonteinspruit Catchment Area (WCA) and covers an area of approximately 86 km^2^. It lies between 26°18′ S and 26°26′ S latitude and 27°23′ E–27°31′ E longitude. Gold exploration in the area dates back to 1898 and mining progressed from 1945 until the present [10]. Geologically, the area is created mainly from sedimentary rocks that consist of banded ironstones, quartzites, tillites, conglomerates, mudstones, and some marine lava deposits [11]. Mining activities are engaged in both deep-level (500 m–4000 m), high-grade underground mining as well as low-grade, surface rock dump mining.

The gold mine has five mine tailings that have been accumulated throughout the operating history of the mine. The topography of the area is relatively flat and the vegetation is largely grassland. The soils are generally sandy loam with livestock farming widespread in the surrounding area [12]. The climate is temperate, with temperatures averaging 24 °C in summer and 13 °C in winter, occasionally dipping below the freezing point. Annual rainfall is about 750 mm [10]. The study area has a population of more than 14,000. There are also some informal settlements residing close to the mine tailings.

### 2.2. Sampling and Measurement of Activity Concentrations

A total of 66 soil samples, 56 from five mine tailings in the study area and 10 from the control area, were randomly collected with a coring tool at a depth of 5 cm. A Global Positioning System (GPS) receiver was used to locate the sampling points. These soil samples were then packaged in plastic bags carrying identification marks according to the IAEA [13]. They were then taken to the laboratory for preparation before analysis. At the laboratory, each sample was dried, crushed into fine power, and mixed to form a homogenous sample. The samples were then transferred and sealed into plastic Marinelli beakers for about four weeks to attain secular equilibrium with some progenies of ^238^U and ^232^Th.

Measurement of activity concentrations in soil samples was carried out by means of a broad-energy germanium (BEGe) detector (BE6530) manufactured by Canberra Industries (Meriden, CT, USA). It has a relative efficiency of 60% and a resolution of 2.0 keV for 1332 keV gamma ray emission of ^60^Co [14]. Energy and efficiency calibrations of the gamma spectrometer were also performed before sample measurement. The activity concentrations of ^238^U, ^232^Th, and ^40^K were calculated based on the weighted average values of their respective daughter products in secular equilibrium. In this analysis, ^238^U was considered as it accounted for its daughter products, such as ^226^Ra.

At the laboratory, the soil samples were first spread out on a plastic sheet and allowed to air dry for 2–3 days. The soil samples were then heated in an electric oven at 110 °C for up to 24 h to remove moisture content and, thereafter, put in an electric furnace at 350 °C for 48 h to burn the plant remains. The samples were then grinded and sieved with a 2 mm stainless steel mesh. The samples, with known masses, were then packed and sealed in plastic Marinelli beakers for 28 days in order to establish a secular equilibrium among some progenies of ^238^U and ^232^Th series. To measure the activity concentrations in soil samples, Marinelli beakers were then placed on top of the detector that was lead shielded to avoid background radiation. The measuring process and analysis of spectra was done using GENIE 2000 software. More information on measurement procedure can be found in our previous paper [10].

### 2.3. RESRAD-OFFSITE Computer Code

Environmental issues are quite challenging to solve because of the complex relationships among the many variables that exist [15]. Modelling, therefore, plays a pivotal role in this regard. One of the most frequently used modelling software is called RESRAD code. RESRAD was developed by the Argonne National Laboratory under the U.S. Department of Energy and the U.S. Nuclear Regulatory Commission as a multifunctional tool to assist in developing criteria for evaluating human radiation doses and risks associated with exposure to radiological contamination [9]. In this evaluation procedure, RESRAD uses U.S. Federal Guidance Report No. 12 [16] and the ICRP-38 radionuclide database. The radionuclide database also accounts for ingrowth of daughters from initially-present parent radionuclides [9]. RESRAD allows users to specify the features of their site and to predict the dose received by an individual at any time up to 100,000 years. It models a site through the use of more than 150 variables. These parameters each has a default value assigned by the developers at Argonne, but can be changed to suit site-specific needs [17].

RESRAD-OFFSITE is an extension of the RESRAD-ONSITE computer code that was developed to estimate the radiological consequences to individuals located onsite or outside the area of primary contamination. The total primary area contaminated by the mine tailings was estimated to be 16 km^2^ and is assumed to be a uniformly distributed layer of soil. The code considers the release of radionuclides from the primary contamination source to the atmosphere, to surface runoff, and to groundwater. It calculates the radiation dose and excess cancer risk with the predicted radionuclide concentrations in the environment [18]. In this study, five exposure pathways were considered in RESRAD-OFFSITE: direct exposure from contamination in soil, inhalation of dust/radon, ingestion of plant foods (crops), ingestion of water (borehole water), and incidental ingestion of soil. By selecting the different pathways, RESRAD-OFFSITE can be used to model the resident exposure scenario [19].

### 2.4. Transport Pathways Associated with NORMs in the Study Area

Major mechanisms of radionuclides transport from potential primary contamination mining sources are the atmosphere, ground water sources, and surface water bodies. Gold mine tailings in the study area constitute a major source of NORMs pollution to the environment. Other sources are rock dumps, processing plants, return-water dams, storm water drainage systems, settling ponds, and evaporation dams. Radionuclides from these sources can either be leached into the underlying ground water aquifers or dissolved and drained through runoff into surface water bodies, thereby contaminating water sources.

**Figure 1:**
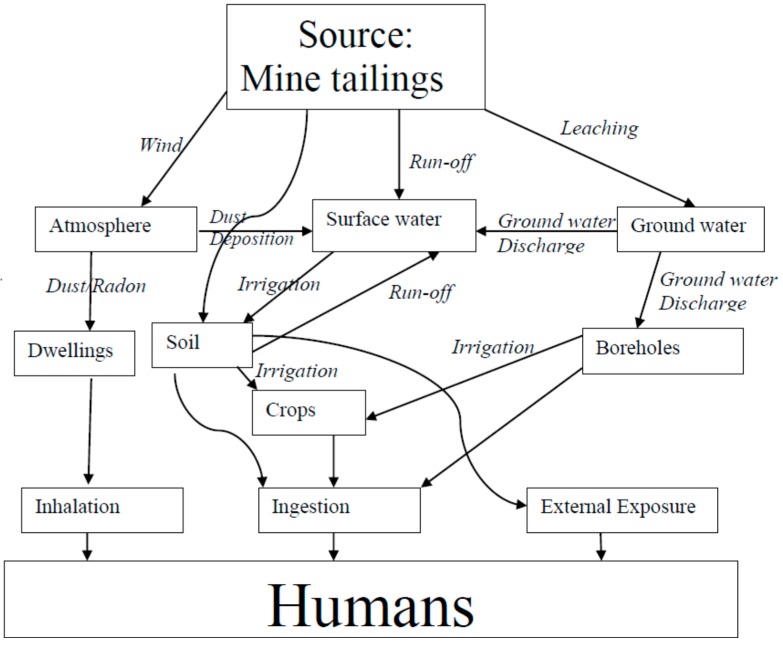
Conceptual site model for the study area.

In some instances, fissure water is treated in water purification plants and used for human consumption. Fissure water comes from underground mine workings and can be a source of contamination if it is in contact with oxidized ore. Water used in mining processes is called process water and usually contains dissolved pollutants. If it is discharged into the environment through either evaporation, seepage, or run-off, it can eventually affect humans. Storm water run-off from areas such as ore piles, gold plants, mine tailings, rock dumps, and return dams can contaminate the environment. In addition, these radionuclides from the primary sources can also be transported to humans via the atmosphere, either as windblown dust or radon. Figure 1 shows the Conceptual Site Model for the Study Area.

### 2.5. Input Parameters and Scenario Description for RESRAD-OFFSITE Code

For the study area, a number of parameters were considered as inputs to the RESRAD-OFFSITE code. The resident scenario was chosen as the critical receptor in this risk assessment. The parameter values were carefully selected to achieve a more realistic estimation of the dose or risk. Where necessary, site-specific parameters replaced default parameters [20].

Input parameters to the model included the measured activity concentrations of ^238^U, ^232^Th, ^40^K, and meteorological data (annual wind speed and direction). Hydrogeological parameters for the various zones were also taken into consideration. These included hydraulic conductivity and density of the soil. Some hydrological parameters, such as total porosity, field capacity, irrigation rate, erosion rate, precipitation rate, runoff coefficient, and soil-specific exponential parameters were also estimated based on the condition of the study area [20]. The area and thickness of the different zones were also taken into account.

The main exposures in this scenario included direct exposure to the radionuclides in soil, inhalation of dust, and ingestion of contaminated vegetables, water, and soil. Dietary information on vegetables, water, and soil were also considered through regulatory guidelines set by the National Nuclear Regulator [21]. Inhalation rate, soil ingestion rate, and the distribution coefficient for radionuclides in the study area were also taken into account according to guidelines [21]. The RESRAD library of radionuclides was then used in this assessment. A summary of the most relevant data used for the risk assessment is shown in Table 1.

## 3. Results and Discussion

### 3.1. NORM Activities Concentration in Soil

Table 2 shows the average activity concentrations of radionuclides with uncertainties for soil samples from the mine tailings and from the control area derived from our earlier paper [10]. These were used as input parameters in RESRAD-OFFSITE code to calculate the radiation dose, as well as the excess cancer morbidity risk.

### 3.2. Radiation Doses Using RESRAD-OFFSITE Computer Code

The total effective dose equivalent (mSv/year) from gold mine tailings and the control area for all of the pathways summed over a duration of 100 years were calculated as shown in Table 3 and Table 4, respectively. According to RESRAD, the maximum TEDE from gold mine tailings during 100 years was found to be 0.0315 mSv/year at year 30 compared to a value of 0.0148 mSv/year from the control area. In both areas, ^232^Th contributed the most to TEDE, while ^40^K and ^238^U had lesser effect.

**Figure 2:**
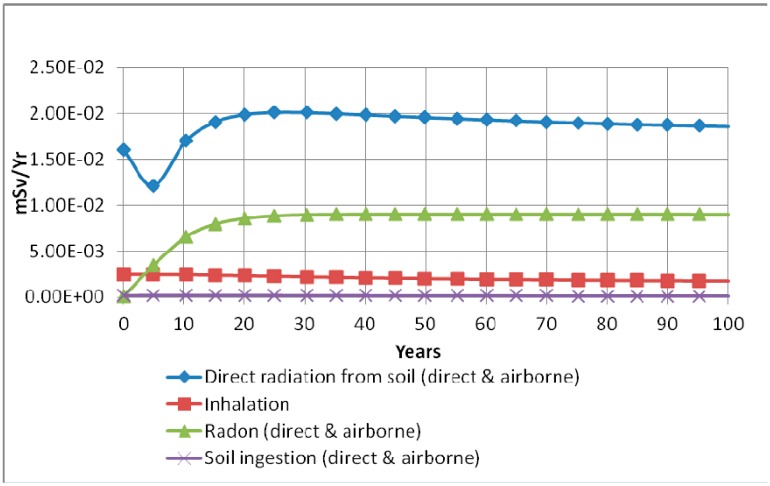
Total effective dose equivalent from all radionuclides summed based on component pathways over a duration of 100 years.

RESRAD-OFFSITE simulation has also shown that the water-independent pathway is the most significant pathway. In this pathway, most of the annual effective dose equivalent is caused by direct radiation from soil followed by radon (direct and airborne), then inhalation, followed by soil ingestion (direct and airborne). Figure 2 shows the radiation doses over a duration of 100 years following contamination.

### 3.3. Excess Cancer Morbidity Risk Using RESRAD-OFFSITE Computer Code

**Figure 3:**
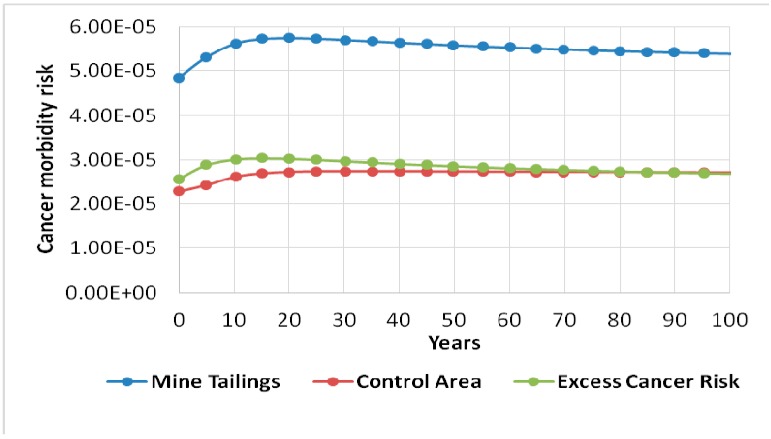
Excess morbidity cancer risks from the gold mine tailings as a result of all radionuclides summed with all pathways summed for the first 100 years.

The results of the radiation doses for the gold mine tailings and control area presented in Table 3 and Table 4 were used by RESRAD-OFFSITE computer code to calculate the excess cancer morbidity risks from for all of the pathways summed over a duration of 100 years. Figure 3 clearly shows the excess cancer morbidity risk from gold mine tailings. This was obtained by subtracting the cancer risk of the control area (assumed to be background) from that of mine tailings. The maximum total excess cancer morbidity risk from gold mine tailings was found to be 3.04 × 10^−5^ at year 15. This progressively decreased to 2.68 × 10^−5^ at year 100. ^232^Th was driving the risk effect followed by ^238^U, and then lastly ^40^K. The US Environmental Protection Agency considers acceptable, for regulatory purposes, a cancer risk in the range of 10^−6^ to 10^−4^ [23]. Therefore, results obtained from the RESRAD-OFFSITE code has shown that the health risk from gold mine tailings is within acceptable levels according to international standards.

### 3.4. Sensitivity Analysis

**Figure 4:**
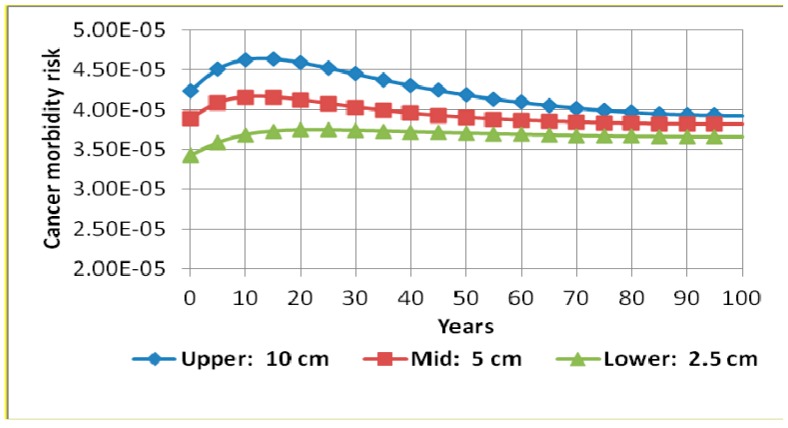
Cancer morbidity risk from all radionuclides (^238^U, ^232^Th, and ^40^K) and all pathways summed during 100 years with sensitivity analysis on contamination zone thickness.

**Figure 5:**
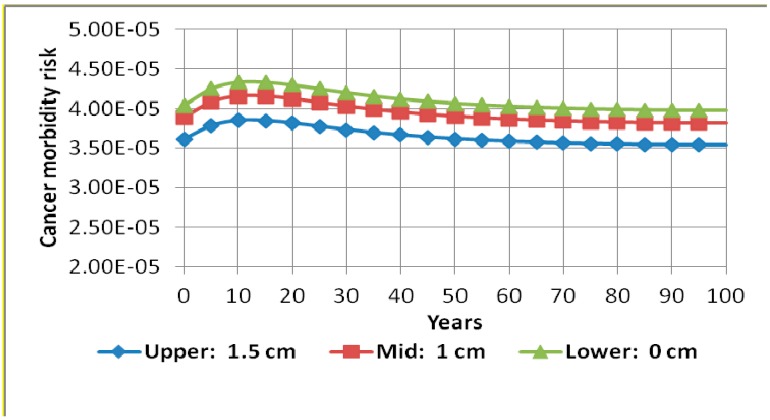
Cancer morbidity risk from all radionuclides (^238^U, ^232^Th, and ^40^K) and all pathways summed during 100 years with sensitivity analysis on cover depth.

Single parameter RESRAD sensitivity analysis on some of the most important parameters was undertaken in order to evaluate the extent of their effect on cancer morbidity risk. These were contamination zone thickness, cover depth, occupancy factor, inhalation rate, soil ingestion rate, and water drinking rate. It was discovered that variations on occupancy factor, inhalation rate, soil ingestion rate, and water drinking rate had insignificant effects on the cancer morbidity risk. However, contamination zone thickness and cover depth affected the results in a significant way. Figure 4 and Figure 5 show results of the sensitivity run on contamination zone thickness and cover depth, respectively. Figure 4 shows that varying the thickness of the contamination zone from 2.5 cm to 10 cm increased the cancer morbidity risk by a maximum of approximately 26%. RESRAD sensitivity analysis has also shown that varying the cover depth from 0 cm to 1.5 cm causes a maximum reduction in cancer morbidity risk of about 13%. This is clearly illustrated in Figure 5.

## 4. Conclusions

This study has evaluated the human risk associated with exposure to NORMs in soils from mine tailings around a gold mine using the RESRAD-OFFSITE code. The total effective dose equivalent (TEDE) from all nuclides was found to range from 1.88 × 10^−2^ to 3.15 × 10^−2^. Results from the study has shown that the water-independent pathway is the most significant pathway contributing to the dose. In this pathway, most of the TEDE is caused by direct radiation from the soil, followed by radon (direct and airborne). The total excess cancer morbidity risk estimated from the model ranged from 2.57 × 10^−5^ to 3.04 × 10^−5^. It has also be noted from the simulation that ^232^Th has a significant contribution to the radiation dose, as well as the excess cancer morbidity risk, compared to ^238^U and ^40^K. This may be attributed to higher dose conversion factors associated with ^232^Th compared to the other two. From the findings of this research it can, therefore, be concluded that gold mine tailings from the study area had a negligible risk in accordance with international guidelines on radiation safety.

## Figures and Tables

**Table 1:** Input Parameters for the RESRAD-OFFSITE code.

Parameter		Value	Description and/or Reference
Average activity concentrations of ^238^U, ^232^Th, and ^40^K		Table 2	The values were based on measurements.
Area of contaminated zone (m^2^)		16,000,000	This was the average estimated over the whole area.
Thickness of contaminated zone (m)		0.05	This was the average estimated over the whole area.
Cover depth (m)		0	There was no cover depth for the radionuclides
Density of contaminated zone (g/m^3^)		1.44	For sandy loam soil [20].
Erosion rate for contaminated zone (m/year)		0.001	RESRAD default value [20].
Total porosity for contaminated zone		0.43	For sedimentary material which is sand fine [20].
Field capacity for contaminated zone		0.2	RESRAD default value [20].
Hydraulic conductivity of contaminated zone (m/year)		1090	For sandy loam soil [20].
Soil-specific exponential b parameter		4.9	For sandy loam soil [20].
Average annual wind speed (m/s)		5	For the area [22].
Evapotranspiration coefficient		0.5	RESRAD default value [21].
Precipitation rate (m/year)		1	The area is relatively humid [21].
Irrigation rate		0.2	RESRAD default value [21].
Runoff coefficient		0.65	For moderately steep, residential area.
Inhalation rate (m^3^/year)		8059.2	For South Africa [21].
Exposure duration (year)		30	RESRAD default value [20].
Fraction of time spent onsite	Indoor	0	For South Africa [21].
	Outdoor	0.1	
Fraction of time spent offsite	Indoor	0.8	For South Africa [21].
	Outdoor	0.1	
Soil ingestion rate (g/year)		37	For South Africa [21].
Leach rate		0	RESRAD default value [20].
Water drinking rate (L/year)		600	For South Africa [21].
Distribution coefficient (K_d_)-uranium (cm^3^/g)		200	For South Africa [21].
Distribution coefficient (K_d_)-thorium (cm^3^/g)		60,000	RESRAD default value [20].
Distribution coefficient (K_d_)-potassium (cm^3^/g)		5.5	RESRAD default value [20].

**Table 2:** Activity concentrations of ^238^U, ^232^Th, and ^40^K for soil samples from the mine tailings [10].

Location	No. of Samples	Parameter	Activity Concentrations (Bq∙kg^−1^)		
			^238^U	^232^Th	^40^K
Control area	10	Average	17.0 ± 0.4	22.2 ± 0.5	496.8 ± 15.2
		Min	12.5 ± 0.3	16.6 ± 0.4	424.3 ± 13.0
		Max	23.6 ± 0.5	30.4 ± 0.6	648.4±19.8
Tailings one	11	Average	733.4 ± 12.7	41.3 ± 0.9	339.9 ± 10.7
		Min	304.4 ± 5.4	36.5 ± 0.8	271.5 ± 9.0
		Max	1243.4 ± 21.6	54.5 ± 1.2	468.4 ± 14.6
Tailings two	13	Average	794.9 ± 13.8	44.9 ± 1.0	460.7 ±14.4
		Min	616.6 ± 10.7	38.4 ± 0.9	226.5 ±7.8
		Max	1391.8 ± 24.1	49.5 ± 1.1	681.9 ± 21.0
Tailings three	8	Average	1556.2 ± 27.0	59.0 ± 1.3	354.3 ± 11.4
		Min	390.9 ± 6.8	22.3 ± 0.6	233.8 ± 7.5
		Max	2668.9 ± 46.2	89.7 ± 1.9	497.1 ± 15.6
Tailings four	12	Average	232.0 ± 4.1	33.2 ± 0.8	489.5 ± 14.1
		Min	87.2 ± 1.6	20.5 ± 0.6	257.9 ± 8.3
		Max	618.2 ± 10.8	49.1 ± 1.1	781.0 ± 23.9
Tailings five	12	Average	609.7 ± 10.6	41.1 ± 0.9	490.7 ± 15.2
		Min	236.1 ± 4.2	25.3 ± 0.6	281.2 ± 8.8
		Max	2054.7 ± 35.6	67.1 ± 1.4	574.9 ± 17.7
Average all tailings			485.3 ± 13.7	43.9 ± 1.0	427.0 ± 13.1

**Table 3:** Total effective dose equivalent (mSv/year) from gold mine tailings for all the pathways summed over a duration of 100 years.

Year	Total Effective Dose Equivalent (mSv/Year)			
	Nuclides			Total
	^40^K	^232^Th	^238^U	
0	1.20 × 10^−2^	2.29 × 10^−3^	4.53 × 10^−3^	1.88 × 10^−2^
5	4.43 × 10^−4^	1.37 × 10^−2^	4.12 × 10^−3^	1.83 × 10^−2^
10	1.18 × 10^−5^	2.25 × 10^−2^	3.72 × 10^−3^	2.62 × 10^−2^
15	4.35 × 10^−7^	2.63 × 10^−2^	3.39 × 10^−3^	2.96 × 10^−2^
20	1.61 × 10^−8^	2.79 × 10^−2^	3.08 × 10^−3^	3.10 × 10^−2^
25	5.94 × 10^−10^	2.86 × 10^−2^	2.81 × 10^−3^	3.14 × 10^−2^
30	1.58 × 10^−11^	2.89 × 10^−2^	2.53 × 10^−3^	3.15 × 10^−2^
35	5.84 × 10^−13^	2.90 × 10^−2^	2.31 × 10^−3^	3.14 × 10^−2^
40	2.16 × 10^−14^	2.91 × 10^−2^	2.10 × 10^−3^	3.12 × 10^−2^
45	7.98 × 10^−16^	2.91 × 10^−2^	1.91 × 10^−3^	3.10 × 10^−2^
50	2.95 × 10^−17^	2.91 × 10^−2^	1.74 × 10^−3^	3.08 × 10^−2^
55	7.83 × 10^−19^	2.90 × 10^−2^	1.57 × 10^−3^	3.06 × 10^−2^
60	2.90 × 10^−20^	2.90 × 10^−2^	1.43 × 10^−3^	3.05 × 10^−2^
65	1.07 × 10^−21^	2.90 × 10^−2^	1.30 × 10^−3^	3.03 × 10^−2^
70	3.96 × 10^−23^	2.90 × 10^−2^	1.18 × 10^−3^	3.02 × 10^−2^
75	1.05 × 10^−24^	2.90 × 10^−2^	1.07 × 10^−3^	3.00 × 10^−2^
80	3.89 × 10^−26^	2.89 × 10^−2^	9.73 × 10^−4^	2.99 × 10^−2^
85	1.44 × 10^−27^	2.89 × 10^−2^	8.86 × 10^−4^	2.98 × 10^−2^
90	5.31 × 10^−29^	2.89 × 10^−2^	8.07 × 10^−4^	2.97 × 10^−2^
95	1.41 × 10^−30^	2.89 × 10^−2^	7.28 × 10^−4^	2.96 × 10^−2^
100	5.22 × 10^−32^	2.88 × 10^−2^	6.63 × 10^−4^	2.95 × 10^−2^

**Table 4:** Total effective dose equivalent (mSv/year) from the control area for all the pathways summed over a duration of 100 years.

Year	Total Effective Dose Equivalent (mSv/Year)			
	Nuclides			Total
	^40^K	^232^Th	^238^U	
0	1.39 × 10^−2^	1.16 × 10^−3^	1.60 × 10^−4^	1.53 × 10^−2^
5	5.15 × 10^−4^	6.92 × 10^−3^	1.60 × 10^−4^	7.60 × 10^−3^
10	1.37 × 10^−5^	1.14 × 10^−2^	1.60 × 10^−4^	1.15 × 10^−2^
15	5.06 × 10^−7^	1.33 × 10^−2^	1.60 × 10^−4^	1.34 × 10^−2^
20	1.87 × 10^−8^	1.41 × 10^−2^	1.60 × 10^−4^	1.43 × 10^−2^
25	6.91 × 10^−10^	1.45 × 10^−2^	1.59 × 10^−4^	1.46 × 10^−2^
30	1.84 × 10^−11^	1.46 × 10^−2^	1.59 × 10^−4^	1.48 × 10^−2^
35	6.79 × 10^−13^	1.47 × 10^−2^	1.59 × 10^−4^	1.48 × 10^−2^
40	2.51 × 10^−14^	1.47 × 10^−2^	1.59 × 10^−4^	1.49 × 10^−2^
45	9.28 × 10^−16^	1.47 × 10^−2^	1.59 × 10^−4^	1.49 × 10^−2^
50	3.43 × 10^−17^	1.47 × 10^−2^	1.59 × 10^−4^	1.49 × 10^−2^
55	9.12 × 10^−19^	1.47 × 10^−2^	1.59 × 10^−4^	1.48 × 10^−2^
60	3.37 × 10^−20^	1.47 × 10^−2^	1.58 × 10^−4^	1.48 × 10^−2^
65	1.25 × 10^−21^	1.47 × 10^−2^	1.58 × 10^−4^	1.48 × 10^−2^
70	4.60 × 10^−23^	1.47 × 10^−2^	1.58 × 10^−4^	1.48 × 10^−2^
75	1.22 × 10^−24^	1.46 × 10^−2^	1.58 × 10^−4^	1.48 × 10^−2^
80	4.52 × 10^−26^	1.46 × 10^−2^	1.58 × 10^−4^	1.48 × 10^−2^
85	1.67 × 10^−27^	1.46 × 10^−2^	1.58 × 10^−4^	1.48 × 10^−2^
90	6.18 × 10^−29^	1.46 × 10^−2^	1.58 × 10^−4^	1.48 × 10^−2^
95	1.64 × 10^−30^	1.46 × 10^−2^	1.57 × 10^−4^	1.48 × 10^−2^
100	6.07 × 10^−32^	1.46 × 10^−2^	1.57 × 10^−4^	1.47 × 10^−2^
